# Epithelial Downgrowth Mixed With Fibrous Ingrowth After Complicated Cataract Surgery: A Challenging Clinical Case

**DOI:** 10.7759/cureus.111571

**Published:** 2026-06-26

**Authors:** Achilleas Mandalos, Dimitrios Tsouris, Paraskevi Markousi, George Kymionis

**Affiliations:** 1 Department of Ophthalmology, General Hospital of Karditsa, Karditsa, GRC; 2 Department of Ophthalmology, General Hospital of Larissa, Larissa, GRC; 3 First Department of Ophthalmology, "G. Gennimatas" Hospital, National and Kapodistrian University of Athens, Athens, GRC

**Keywords:** cataract surgery, corneal transplantation, epithelial downgrowth, fibrous ingrowth, glaucoma

## Abstract

Epithelial downgrowth (ED) is a rare but dreaded complication of intraocular surgery or trauma and is characterized by the intraocular migration or seeding of epithelial cells from the ocular surface, sometimes with concurrent intraocular proliferation of fibrovascular tissue. Due to its insidious development, it may present diagnostic difficulties to the ophthalmologist; moreover, its relentless progression poses a tough therapeutic challenge with a generally poor visual prognosis. In this paper, we describe the case of a 68-year-old man who developed mixed ED and fibrous ingrowth (FI) after complicated cataract surgery. His clinical picture was initially misdiagnosed and treated as malignant glaucoma with only a brief period of improvement. Surgical removal of a specimen of the pathologic intraocular tissue, followed by its cytological analysis, confirmed the diagnosis. Due to intractably high intraocular pressure (IOP) and persistent corneal edema, we proceeded to glaucoma shunt implantation and subsequently to full-thickness corneal transplantation. Eighteen months after the keratoplasty, the patient fares well with a reasonably good vision of 20/30, a clear corneal graft, and no signs of downgrowth recurrence. This case illustrates the diagnostic and therapeutic challenges posed by ED but also the importance of its multidisciplinary management in order to achieve the best possible visual outcome for the patient.

## Introduction

Epithelial downgrowth (ED) is a rare but potentially devastating complication of intraocular surgery or trauma. In this condition, epithelial cells of conjunctival and/or corneal origin are either seeded into the eye during surgery or migrate into the eye through a traumatic or surgical wound, proliferating in the intraocular structures and causing extensive damage to the corneal endothelium, iris surface, and the trabecular meshwork. ED may take the form of free-floating cells, cysts, or a retrocorneal sheet that may extend over the angle and the iris surface. Immunohistochemistry analysis has shown that epithelial cells in ED are mainly of conjunctival rather than corneal origin [1].

Closely associated and sometimes co-existent with ED is fibrous ingrowth (FI), which is characterized by the intraocular proliferation of fibrovascular tissue through a penetrating wound, especially in eyes complicated with prolonged inflammation or intraocular hemorrhage. It is thought that this fibroblast activation is part of the wound repair and scarring process that occurs in the anterior segment of the eye after surgery or injury [2]. Furthermore, in such cases, the inflammatory cells may induce endothelial fibrous metaplasia [3] and abnormal proliferation and migration of iris melanocytes and lens epithelial cells [4,5].

Epithelial and FI result in severe corneal edema due to failure of the endothelial pump, iris distortion, and peripheral anterior synechiae with resultant refractory angle-closure glaucoma. Due to its clinical appearance and its relentless progression, which severely disrupts the normal anatomy and function of the anterior eye segment, it can sometimes mimic other conditions such as malignant glaucoma or persistent inflammation. It requires timely recognition and prompt management.

In this paper, we present the diagnostic and therapeutic challenges of a case of mixed ED/FI that developed insidiously after complicated phacoemulsification surgery in a patient with a previous history of herpes zoster ophthalmicus, was initially treated as persistent intraocular inflammation from potential viral reactivation and later as malignant glaucoma, and was finally managed with glaucoma tube implantation and penetrating keratoplasty with good visual recovery (20/30) 18 months postoperatively. Consent was obtained from the patient to use non-identifiable digital material (images and videos) for the purpose of publication.

## Case presentation

**Figure 1:**
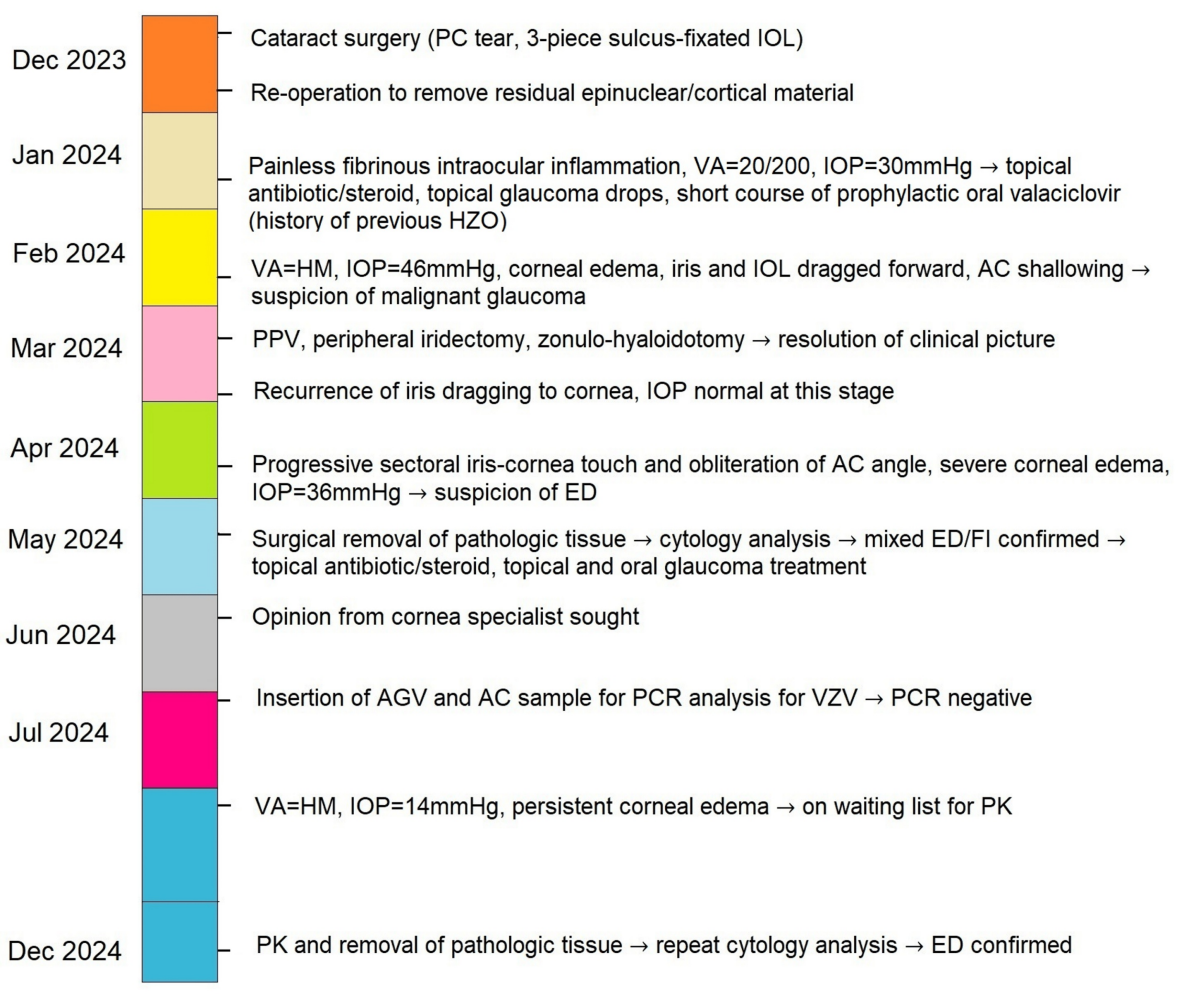
Timeline of clinical course and procedures. PC: posterior capsule; IOL: intraocular lens; VA: visual acuity; IOP: intraocular pressure; HZO: herpes zoster ophthalmicus; AC: anterior chamber; AGV: Ahmed Glaucoma Valve; PCR: polymerase chain reaction; VZV: varicella zoster virus; HM: hand movement; PK: penetrating keratoplasty; ED: epithelial downgrowth; FI: fibrous ingrowth

A 68-year-old male patient underwent complicated cataract surgery with posterior capsule rupture but no vitreous loss and was fitted with a three-piece hydrophobic acrylic sulcus-fixated intraocular lens (IOL) in his right eye in December 2023 (Figure 1).

The main clear cornea incision (placed superiorly) was closed with a 10-0 nylon suture. Two weeks later, he returned to the operating room to remove some residual epinuclear and cortical material that had gradually moved behind the IOL and blocked the visual axis. This reoperation involved a bimanual approach with the creation of two side ports, one made nasally and the other temporally. An additional 10-0 nylon suture was placed at the temporal side-port incision at the conclusion of surgery, and both sutures were removed approximately two weeks later. The postoperative course was uneventful for about four weeks, after which point he returned for a follow-up complaining of foggy vision, and his visual acuity was measured at 20/200. A fibrinous inflammatory reaction with an intraocular pressure (IOP) of 30 mmHg was noted, and he was put on intensive topical antibiotic and steroid treatment as well as topical glaucoma drops. In the absence of ocular pain, redness, or hypopyon, an episode of late-onset endophthalmitis was deemed improbable; therefore, no more drastic action was taken at this point. However, as the patient recalled an episode of herpes zoster ophthalmicus in this eye some years earlier, he was also prescribed a prophylactic one-week course of oral valaciclovir 500 mg three times a day for fear of potential viral intraocular reactivation.

Despite an initial response to the above treatment, clinical deterioration was noted three weeks later with high IOP (IOP: 46 mmHg), severe corneal edema, visual acuity of “hand movement," and a strange dragging of the iris forward towards the cornea at the superior and temporal quadrants, causing significant corectopia, shallowing of the anterior chamber, peripheral iris-cornea touch, and mild forward and nasal IOL dislocation. Malignant glaucoma was suspected as the underlying mechanism, and the patient was referred to a vitreoretinal specialist who proceeded to full vitrectomy, peripheral iridectomy, and zonulo-hyaloidotomy, as well as lysis of any visible peripheral anterior synechiae.

**Figure 2:**
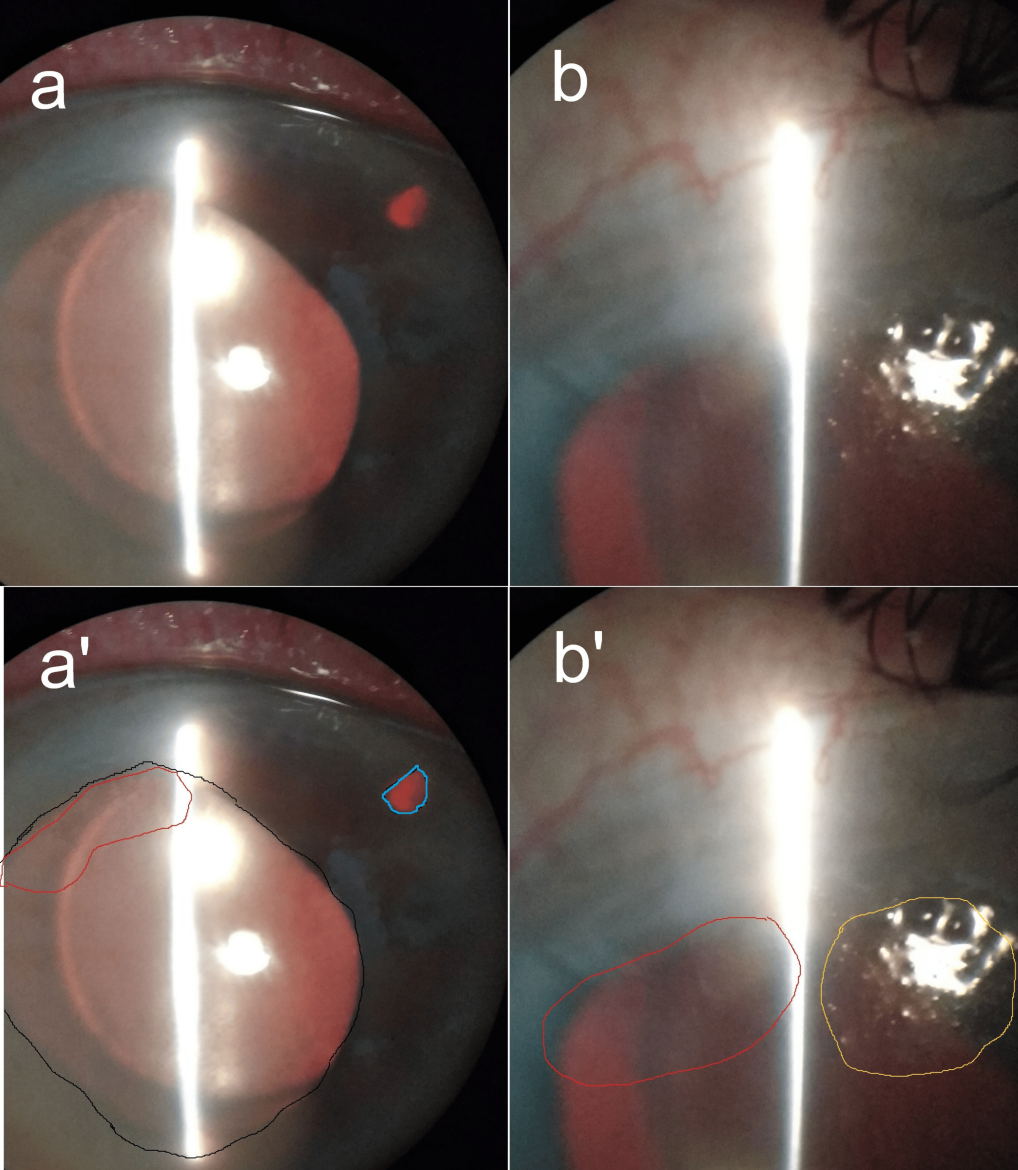
Slit-lamp photographs. Without (a, b) and with (a’, b’) contour annotations of the right eye of the patient showing significant microcystic corneal edema (b’, yellow-contoured area) and excessive dragging of the iris towards the cornea in the superior and temporal quadrants (a’, pupil border contoured in black) caused by a fine membranous tissue developing on the posterior corneal surface and on the iris surface (a’, b’, red-demarcated area). A patent peripheral iridectomy and zonulo-hyaloidotomy created by the vitreoretinal surgeon is noted (a’, blue-contoured).

After a brief period of a few days of overall clinical improvement, the patient returned two weeks later, stating vision deterioration. At this point, a fine membranous tissue on the posterior surface of the superior cornea was noted, which seemed to extend superiorly and temporally and was dragging the iris upwards, actually sticking it to the cornea (Figure 2), also causing corneal edema due to endothelial pump dysfunction.

**Figure 3:**
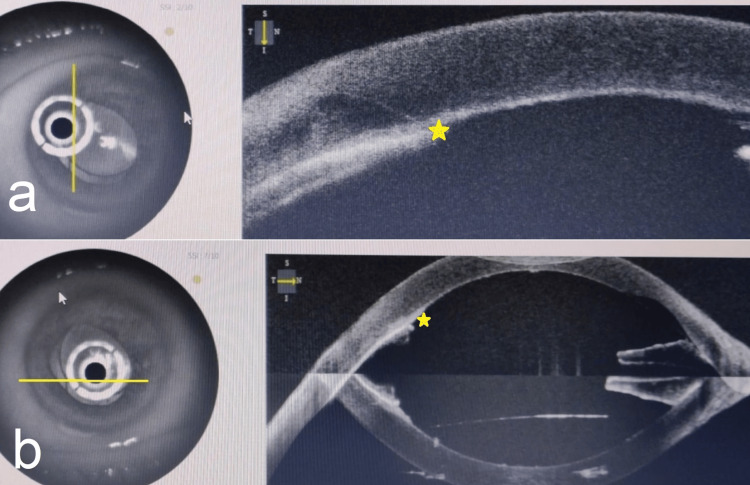
Anterior segment OCT. Showing iris-cornea contact in the superior (a) and temporal (b) quadrants with complete obliteration of the anterior chamber angle. Note the hyperreflective tissue lining the posterior corneal surface (asterisk). OCT: optical coherence tomography

There was no apparent intraocular inflammation, and the IOP was normal. Broad areas of iris-cornea touch in the superior and temporal quadrants were noted on gonioscopy, but the rest of the anterior chamber angle appeared to be open. However, in the course of the following few weeks, it became evident that this membranous tissue gradually expanded and essentially obliterated the temporal half of the anterior chamber angle (Figure 3), aggravating the corneal edema, thus precluding reliable gonioscopy and causing intractably high IOP (36 mmHg).

**Figure 4:**
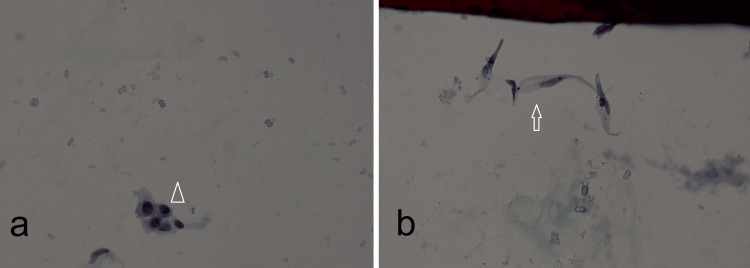
Cytology images. Papanicolaou stain, 400x total magnification from the specimen of pathologic tissue showing a cluster of cuboidal epithelial-like cells (a, open triangle) and spindle-shaped fibroblasts (b, arrow).

At this point, we suspected ED/FI and listed the patient for surgical removal of as much of the pathologic tissue as possible via the already existing superior corneal incision created at the time of the initial cataract surgery, which was then closed with a new 10-0 nylon suture. According to the cytology report, analysis of the retrieved tissue and aqueous sample showed a mixture of fibroblasts, cuboidal epithelial cells, and non-keratinized squamous epithelial cells with a possible origin from the ocular surface, thus corroborating the clinical suspicion (Figure 4). Immunohistochemistry was not performed on the tissue specimen.

An opinion from a cornea expert was sought, and the decision was made to proceed to an anterior chamber tap for polymerase chain reaction (PCR) analysis for varicella zoster virus (VZV) and surgical management of the intractably high IOP with the insertion of an Ahmed Glaucoma Valve (AGV) and then to full-thickness corneal transplantation in the hope of restoring some useful vision despite the poor prognosis of such cases. PCR analysis returned negative results for VZV, and the insertion of an AGV by a glaucoma specialist did manage to effectively control IOP. Of note, despite the prolonged duration of high IOP, the optic disc of our patient did not show any signs of glaucoma, and the AGV implantation served both to prevent the development of glaucomatous damage and to facilitate the planned corneal transplantation.

Corneal transplantation followed a few months later (Video 1), during which further membranous tissue removal was also performed, and the specimen was sent for repeat cytology analysis, which confirmed the presence of squamous epithelial and goblet cells. During the operation, a deep suture track at the temporal corneal incision was recognized as the potential entry site for epithelial cells and was closed securely with tightly spaced sutures.

**Video 1:** Penetrating keratoplasty (performed by GK). Involved removal of as much of the pathologic tissue as possible via an open-sky approach, mechanical 360° opening of the anterior chamber angle, and shortening of the intraocular portion of the glaucoma tube in order to avoid contact with the corneal graft. The graft was secured in place with interrupted 9-0 nylon sutures.

**Figure 5:**
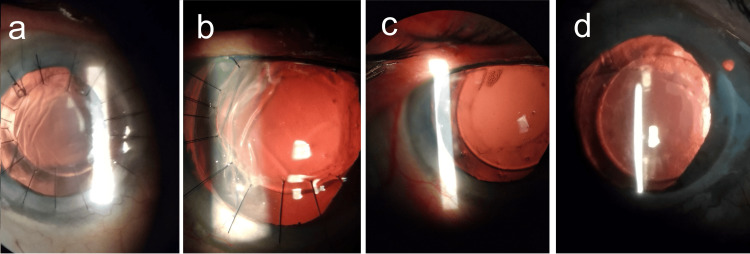
Slit-lamp photographs. Showing the postoperative course after penetrating keratoplasty at approximately the following time points: three months (a), six months (b), 12 months (c), 18 months (d).

Postoperatively, the patient remained on long-term intensive topical steroids and prophylactic oral antiviral treatment with valaciclovir. His postoperative course was uneventful, his vision showed signs of gradual improvement, and his IOP remained normal. Most importantly, he did not show any signs of ingrowth relapse on the corneal graft (Figure 5). At his most recent appointment, 18 months postoperatively, his best-corrected visual acuity was 20/30, the corneal graft was clear, IOP was 14 mmHg, and the optic disc remained normal.

## Discussion

This case underscores the diagnostic and therapeutic challenges posed by ED/FI with insidious onset. We hypothesize that a tight and excessively deep suture at the temporal corneal incision during the reoperation for removal of residual lens material may have acted as a fistula giving access to the intraocular space to epithelial cells from the ocular surface. Fibroblast reaction was probably secondary to the epithelial migration into the anterior chamber and the prolonged postoperative inflammatory period. Due to the subtle nature of ED/FI development, the clinical picture was initially misattributed to an aqueous misdirection-like mechanism involving high posterior pressure and anterior displacement of the iris-IOL diaphragm with subsequent angle closure, high IOP, and corneal edema.

Various risk factors for ED have been recognized, including multiple intraocular surgeries, poor wound closure [6], wound fistulas [7], iris or vitreous incarceration [8], longstanding inflammation, and full-thickness sutures [9,10]. It seems that in our patient, quite a few of the above risk factors were present. Still, ED occurs rarely, with an incidence of between 0.076 and 0.12% after cataract surgery in the pre-phacoemulsification era as reported in a study published in the ’80s [11], although it has been described even after uneventful clear-cornea phacoemulsification [6,8,9]. Therefore, the surgeon should remain vigilant for suspicious signs of this condition, as these can initially be rather subtle.

Laboratory diagnosis of ED is set by histopathologic [11] or cytology [12] analysis, which shows non-keratinized squamous epithelial cells originating from the ocular surface, while immunohistochemical analysis using specific markers may help identify the corneal or conjunctival origin of the invading epithelial cells [1]. In our case, we confirmed the diagnosis by fine needle aspiration cytology analysis conducted in two different instances (at the operation to remove the pathologic tissue and at the time of corneal transplantation) by two independent laboratories, both reporting the presence of cuboidal/goblet cells and non-keratinized squamous epithelial cells.

Treatment for ED is challenging, with a guarded prognosis for vision and high recurrence rates. Treatment may include membrane excision and fistula repair or even en bloc excision of the involved tissue and tectonic graft [13,14] along with local cryotherapy [9]. Other treatment modalities include intracameral injection of 5-fluorouracil and laser photocoagulation of the pathologic tissue [15,16]. Long-standing corneal edema requires corneal transplantation, either penetrating keratoplasty (PK) [7,9,17] or Descemet membrane endothelial keratoplasty (DMEK) [6,18]. Final visual acuity after treatment for ED varies widely from 20/200 [13,17] to 20/50 [18] or even 20/30 [9].

## Conclusions

This case illustrates the potential benefit of timely suspicion, tissue confirmation, IOP control, and cornea rehabilitation in selected patients. In fact, our patient required a rather aggressive approach with glaucoma shunt implantation to control IOP and PK with concurrent removal of the pathologic ingrowth tissue in order to restore anterior segment anatomy and clear refractive media. Thankfully, owing to the multidisciplinary expert management of the condition and despite the grave prognosis of such cases, our patient recovered good visual function. The diagnosis of ED/FI should be suspected in case of otherwise unexplained iris distortion and dragging towards the cornea and persistent corneal edema. Furthermore, its management requires thorough removal of the abnormal tissue in order to reduce the risk of recurrence, identification and closure of the fistula that acts as an entry site for the epithelial cells, and management of any sequelae such as high IOP and corneal decompensation. Lastly, it is important to remain vigilant for recurrences even after many years; therefore, long-term surveillance is recommended.
